# Clinical, Functional, and Comorbid Characteristics of COPD Patients with Impaired Diffusing Capacity: A Cross-Sectional Study

**DOI:** 10.3390/jcm15103861

**Published:** 2026-05-17

**Authors:** Linlin Tang, Yu Jiang

**Affiliations:** 1 The Fourth Clinical College, Chongqing Medical University, Chongqing 400016, China; 2023122262@stu.cqmu.edu.cn; 2 University-Town Hospital of Chongqing Medical University, Chongqing Medical University, Chongqing 400016, China

**Keywords:** diffusing capacity of the lung for carbon monoxide (DLCO), chronic obstructive pulmonary disease (COPD), clinical characteristics, risk stratification, prognosis

## Abstract

**Background:** The diffusing capacity of the lung for carbon monoxide (DLCO) is a key measure of alveolar–capillary gas exchange, but its clinical significance in chronic obstructive pulmonary disease (COPD) remains incompletely defined. This study aimed to characterize the demographic, clinical, functional, and comorbid profiles of COPD patients stratified by the degree of DLCO impairment, and to evaluate the potential value of DLCO as a marker for disease severity and clinical phenotyping in a Chinese cohort. **Methods:** This single-center retrospective cross-sectional study enrolled 650 patients diagnosed with COPD (according to GOLD 2025 criteria) who underwent pulmonary function tests between January 2024 and February 2025 at the university-town hospital of Chongqing Medical University. Patients were stratified by predicted DLCO% into four groups: normal (≥80%), mild impairment (60–79%), moderate impairment (40–59%), and severe impairment (<40%). Demographic, clinical, laboratory, pulmonary function, echocardiographic, and chest CT data were collected. Comparisons across groups were performed using ANOVA/Kruskal–Wallis tests, chi-square or Fisher’s exact tests, and Spearman correlation analysis (IBM SPSS Statistics 25.0). Due to the exploratory nature of the study, no adjustment for multiple comparisons was applied. **Results:** Progressive DLCO impairment was associated with a higher proportion of male patients (69.2% to 90.9%, *p* = 0.018), older age (67.3 ± 9.0 to 72.9 ± 6.7 years, *p* < 0.001), lower BMI (median from 23.9 to 20.0 kg/m^2^, *p* < 0.001), and higher smoking prevalence (58.7% to 87.5%, *p* = 0.001). The prevalence of pulmonary tuberculosis rose markedly (0.58% to 9.09%, *p* = 0.037). All spirometric parameters declined (e.g., FEV1%pred from 67.3% to 32.6%, *p* < 0.001). Systemic inflammatory markers (NLR, SII) increased, while hemoglobin and albumin decreased (both *p* < 0.001). Respiratory failure occurred in 30.0% of the severe DLCO group (predominantly type I, *p* <0.001). Echocardiography revealed a decline in left ventricular ejection fraction (61.2 ± 5.0% to 59.1 ± 4.0%, *p* = 0.012) and a trend toward higher pulmonary hypertension risk (27.8%, *p* = 0.056). DLCO%pred correlated positively with FEV1%pred (r = 0.394, *p* < 0.001) and oxygen saturation (r = 0.151, *p* < 0.001), and negatively with NLR (r = −0.165, *p* < 0.001) and SII (r = −0.149, *p* < 0.001). **Conclusions:** In COPD, DLCO impairment is associated with distinct clinical phenotypes, including male sex, advanced age, malnutrition, increased tuberculosis risk, worse lung function, systemic inflammation, and respiratory/cardiac dysfunction. These findings support DLCO as a valuable complementary marker for disease severity characterization in COPD. Longitudinal studies are needed to confirm its prognostic value.

## 1. Introduction

Chronic obstructive pulmonary disease (COPD) is a leading cause of morbidity and mortality worldwide, characterized by persistent airflow limitation and heterogeneous clinical presentations [1]. Although forced expiatory volume in 1 s (FEV1) remains the cornerstone of COPD assessment, it inadequately captures the disease’s complexity—including alveolar-capillary dysfunction, systemic inflammation, and multi-morbidity [2,3]. The diffusing capacity of the lung for carbon monoxide (DLCO) is a key measure of alveolar–capillary gas exchange efficiency and reflects structural damage to the pulmonary parenchyma and microcirculation [4]. Previous studies have linked reduced DLCO to poorer outcomes in COPD, such as increased exacerbation risk, diminished quality of life, and higher mortality [5,6]. However, the specific clinical phenotypes, comorbidity patterns, and multisystem functional correlates of COPD patients with progressive DLCO impairment remain incompletely understood [7,8,9].

Emerging evidence suggests that DLCO decline may signal pathological processes beyond airflow limitation, including emphysema destruction, pulmonary vascular remodelling, and systemic inflammatory dysregulation [10,11]. Moreover, associations between DLCO impairment and certain comorbidities (e.g., pulmonary tuberculosis, pulmonary hypertension) have been reported, but these relationships lack systematic validation in large cohorts [1,12]. Clarifying the clinical characteristics of COPD patients stratified by DLCO severity could enhance risk stratification, guide personalized management, and identify unmet clinical needs.

Therefore, this retrospective study aimed to: (1) delineate the demographic, clinical, and functional profiles of COPD patients with varying degrees of DLCO impairment; (2) investigate the associations between DLCO decline and key outcomes—including comorbidities, systemic inflammation, and respiratory/cardiac dysfunction; and (3) assess the potential value of DLCO as a severity stratification marker in COPD.

## 2. Methods

### 2.1. Study Design and Population

This single-center retrospective cross-sectional study was conducted at the University-Town Hospital of Chongqing Medical University. As this was a hospital-based cohort derived from a pulmonary function laboratory, the study population represents symptomatic COPD patients who were referred for formal lung function testing, rather than a random community sample. Eligible participants were patients diagnosed with COPD according to the GOLD 2025 criteria [1] who underwent pulmonary function tests between January 2024 and February 2025. All patients were enrolled during stable outpatient visits or at the time of hospitalization for routine clinical evaluation; none were in acute exacerbation at the time of pulmonary function testing.

Inclusion criteria were: (1) post-bronchodilator FEV1/FVC < 0.70; (2) complete pulmonary function data including DLCO measurement; and (3) availability of clinical, laboratory, and imaging records.

Exclusion criteria were: (1) previous lobectomy or segmentectomy; (2) massive pleural effusion, pneumothorax, or confirmed interstitial lung disease; (3) concurrent bronchial asthma, severe thoracic or spinal deformities, or cognitive/physical impairment precluding data collection; or (4) incomplete clinical data.

Patients were stratified into four groups according to predicted DLCO% (DLCO%pred) [11]: normal (≥80%), mild impairment (60–79%), moderate impairment (40–59%), and severe impairment (<40%). Pulmonary function testing was performed using a MIS-PFT System (Germany) with reference equations derived from the Chinese population. DLCO was measured using the single-breath method and was not corrected for hemoglobin levels. The transfer coefficient (KCO) was not available. Given the small number of patients in the severe impairment group (*n* = 33), findings for this subgroup should be interpreted with caution. The study protocol was approved by the Institutional Review Board of Chongqing Medical University; informed consent was waived owing to the retrospective design.

### 2.2. Data Collection

#### 2.2.1. Demographic and Clinical Data

Demographic variables included age, sex, and body mass index (BMI, calculated as weight in kg divided by height in meters squared). Smoking history was categorized as current, former, or never smoker; cumulative exposure was quantified in pack-years (1 pack-year = 20 cigarettes/day for 1 year). Clinical symptoms (cough, expectoration, chest tightness, exertional dyspnoea, fatigue) and physical signs (barrel chest, wheezes, moist rales) were recorded as present or absent based on medical history and physical examination at admission. Comorbidities—including hypertension, type 2 diabetes mellitus, coronary heart disease, pulmonary tuberculosis, and malignant tumours—were ascertained using ICD-10 codes, laboratory results, imaging, and/or pathological reports.

#### 2.2.2. Pulmonary Function Tests

Pulmonary function tests were performed using a standardized desktop spirometer (Model:Jaeger MasterScreen PFT System, Bavaria, Germany) with a DLCO module, in strict accordance with the ATS/ERS 2005 and 2019 guidelines for lung function testing [13,14]. All tests were completed by certified respiratory therapists in the hospital’s pulmonary function laboratory, and each patient completed at least 3 valid spirometry maneuvers (meeting ATS/ERS acceptability and repeatability criteria). Key measured indices included:

1. Ventilatory function: FEV1%pred, FVC%pred, vital capacity (VC%pred), maximal mid-expiratory flow (MMEF%pred), maximum expiratory flow at 75%, 50%, and 25% of FVC (MEF75%pred, MEF50%pred, MEF25%pred);
2. Diffusion function: DLCO (single-breath method) and DLCO%pred (calibrated for Chinese reference values). DLCO values were not corrected for hemoglobin levels, which may influence interpretation given the significant differences in hemoglobin levels across groups. Alveolar volume (VA) and the transfer coefficient (KCO) were not available in this retrospective dataset, limiting our ability to distinguish between alveolar loss and membrane dysfunction.

#### 2.2.3. Laboratory and Imaging Assessments

Serological indices included: (1) Blood routine (white blood cell count, hemoglobin, neutrophil/lymphocyte ratio (NLR)); (2) Systemic immune-inflammation index (SII = platelet count × neutrophil count/lymphocyte count); (3) Arterial blood gas analysis (PaO_2_, PaCO_2_, oxygen saturation); and (4) Albumin, C-reactive protein, and NT-proBNP levels.

Echocardiography was performed at the discretion of the attending physician, typically in patients with suspected cardiac dysfunction or unexplained dyspnea. Echocardiographic parameters (left ventricular ejection fraction (LVEF), right ventricular outflow tract diameter, pulmonary arterial systolic pressure (PASP)) were recorded. Chest CT findings (emphysema type, pulmonary fibrosis features, bronchiectasis, bullae) were independently reviewed by two radiologists and one respiratory physician; discrepancies were resolved via consensus.

### 2.3. Statistical Analysis

Statistical analyses were performed using IBM SPSS Statistics 25.0. Normality of continuous variables was tested via the Shapiro–Wilk test. Normally distributed data are presented as mean ± standard deviation (SD) and compared using one-way ANOVA; non-normally distributed data are expressed as median (interquartile range (IQR)) and analyzed via Kruskal–Wallis H test. Categorical variables are presented as frequencies (percentages) and compared using chi-square test or Fisher’s exact test (for small expected values). Spearman correlation was used to assess associations between DLCO and continuous variables.

Multivariable logistic regression was performed to identify factors independently associated with DLCO impairment, adjusting for age, sex, BMI, smoking status, and FEV_1_%pred. Missing data were not imputed; the number of available cases is indicated in each table. Missingness was due to incomplete clinical records or unavailable test results. Given the exploratory nature of the study, no formal adjustment for multiple comparisons was applied; thus, findings should be interpreted with caution. A two-tailed *p* < 0.05 was considered statistically significant.

## 3. Results

### 3.1. Baseline Demographic and Clinical Characteristics

A total of 650 COPD patients were included, with 172 (26.5%) in the Normal DLCO group, 256 (39.4%) in the Mild impairment group, 188 (28.9%) in the Moderate impairment group, and 33 (5.1%) in the Severe impairment group. As the severe DLCO group comprised only 33 patients, findings for this subgroup, particularly for comorbidities with low event counts, should be interpreted with caution.

As DLCO impairment worsened, the proportion of male patients increased progressively (from 69.2% in the normal group to 90.9% in the severe group, *p* = 0.018), as did age (mean increased from 67.3 ± 9.0 to 72.9 ± 6.7 years, *p* < 0.001). Median BMI declined significantly across groups (from 23.9 to 20.0 kg/m^2^, *p* <0.001). Smoking prevalence also rose with DLCO severity (58.7% to 87.5%, *p* = 0.001), whereas pack-years did not significantly differ (*p* = 0.169).

In terms of symptoms, shortness of breath (73.8% vs. 76.2% vs. 80.9% vs. 93.9%, *p* = 0.047) and chest tightness (5.2% vs. 13.2% vs. 8.0% vs. 3.0%, *p* = 0.017) showed significant group differences. Barrel chest (36.0% vs. 37.7% vs. 42.6% vs. 69.7%, *p* = 0.003) and wheezes (29.1% vs. 17.9% vs. 22.9% vs. 36.4%, *p* = 0.014) were more prevalent in the severe DLCO impairment group (Table 1).

### 3.2. Comorbidity Profiles

Pulmonary tuberculosis prevalence increased significantly with DLCO impairment (0.58% vs. 2.73% vs. 3.19% vs. 9.09%, *p* = 0.037). While hypertension, diabetes mellitus, and coronary heart disease showed no group differences (all *p* > 0.05), the proportion of confirmed lung cancer (3.49% vs. 6.61% vs. 6.38% vs. 12.12%, *p* = 0.227) and other tumors (4.65% vs. 3.52% vs. 5.85% vs. 9.09%, *p* = 0.431) trended higher in the severe DLCO impairment group (Table 2). These non-significant trends should be interpreted with caution due to small numbers.

### 3.3. Pulmonary Function Indices

All pulmonary function parameters showed a progressive decline with DLCO impairment (all *p* < 0.01). FEV1%pred decreased from 67.3% (IQR: 52.7, 84.4) in the Normal group to 32.6% (IQR: 23.5, 51.7) in the Severe group (*p* < 0.001). Similarly, FVC%pred (87.7% vs. 86.9% vs. 79.2% vs. 64.1%, *p* < 0.001), VC%pred (87.5% vs. 84.9% vs. 77.6% vs. 64.3%, *p* < 0.001), and MMEF%pred (23.7% vs. 18.7% vs. 15.5% vs. 9.7%, *p* < 0.001) declined significantly. Small airway function indices (MEF75%, MEF50%, MEF25%) also showed consistent decreases across groups (Table 3).

### 3.4. Serological and Cardiac Function Results

Serological analysis showed that hemoglobin (140.0 vs. 130.0 vs. 130.0 vs. 126.5 g/L, *p* < 0.001) and albumin (41.6 vs. 40.1 vs. 39.2 vs. 39.1 g/L, *p* < 0.001) levels decreased with DLCO impairment, while anemia prevalence increased (5.2% vs. 15.6% vs. 22.9% vs. 30.3%, *p* < 0.001). Systemic inflammatory markers (NLR: 3.9 vs. 4.0 vs. 4.2 vs. 5.4, *p* = 0.002; SII: 778.3 vs. 861.4 vs. 882.6 vs. 1161.1, *p* = 0.010) were significantly higher in the severe DLCO impairment group.

Oxygen saturation declined progressively (95.8% vs. 95.0% vs. 94.3% vs. 93.8%, *p* = 0.018), and respiratory failure incidence increased (6.3% vs. 13.9% vs. 16.6% vs. 30.0%, *p* < 0.001), predominantly type I respiratory failure (Table 4).

Echocardiographic data showed that EF% decreased with DLCO severity (61.2 ± 5.0 vs. 61.9 ± 4.3 vs. 60.1 ± 5.2 vs. 59.1 ± 4.0, *p* = 0.012). Although this difference reached statistical significance, the absolute change was small and may not be clinically meaningful. Right ventricular outflow tract diameter was significantly larger in the Moderate group (27.4 ± 3.3 mm, *p* = 0.041), and pulmonary hypertension prevalence trended higher in the Severe group (27.8%, *p* = 0.056) (Table 5). It should be noted that echocardiography was performed in a non-random subset of patients, potentially introducing selection bias.

### 3.5. Chest CT Findings

As shown in Table 6, comparison of chest CT imaging characteristics among COPD patients with varying degrees of DLCO impairment revealed that the prevalence of several emphysema- and fibrosis-related imaging findings increased significantly with worsening DLCO impairment. The prevalence of both centrilobular emphysema and panlobular emphysema increased significantly with greater DLCO impairment (*p* < 0.001). Notably, centrilobular emphysema was observed in 75.8% of patients in the severe DLCO impairment group, suggesting it is the predominant imaging phenotype associated with declining pulmonary function. Honeycombing and reticulation were more prevalent in the moderate and severe DLCO impairment groups, with significant differences across groups (*p* = 0.010 and *p* = 0.012, respectively), indicating an association between fibrotic changes and reduced diffusing capacity. The prevalence of bullae and traction bronchiectasis also increased with worsening pulmonary function. Bullae were present in 45.5% of patients in the severe DLCO impairment group compared with 13.4% in the normal DLCO group (*p* < 0.001).

### 3.6. Correlation Between DLCO and Clinical Indices

Spearman correlation analysis showed that DLCO%pred was positively correlated with FEV1%pred (r = 0.394, *p* < 0.001), FVC%pred (r = 0.374, *p* < 0.001), oxygen saturation (r = 0.151, *p* < 0.001), and lymphocyte count (r = 0.130, *p* < 0.001). In contrast, DLCO%pred was negatively correlated with NLR (r = −0.165, *p* < 0.001), SII (r = −0.149, *p* < 0.001), and neutrophil percentage (r = −0.146, *p* < 0.001) (Table 7).

### 3.7. Logistic Regression Analysis of Factors Associated with DLCO Function

Given that the DLCO values in our institutional pulmonary function reports were raw measurements without hemoglobin correction, and considering that hemoglobin is a physiological determinant of diffusing capacity—low hemoglobin may lead to falsely reduced DLCO, whereas polycythemia may result in falsely elevated values—we applied the Cotes method to correct DLCO based on hemoglobin levels using the following formula:

Corrected DLCO = uncorrected DLCO × (1.7 × Hb)/(10.22 + Hb)

where Hb is expressed in g/dL. Corrected DLCO values were then used in the subsequent logistic regression analysis.

As shown in Table 8, variables with *p* < 0.05 in univariable analyses were entered into a multivariable logistic regression model with DLCO function (normal = 1, reduced = 0) as the dependent variable. The results revealed that age (OR: 0.968, 95% CI: 0.943–0.993) was an independent risk factor for reduced DLCO function, whereas BMI (OR: 1.183, 95% CI: 1.108–1.263), oxygen saturation (OR: 1.128, 95% CI: 1.043–1.219), and hemoglobin (OR: 1.622, 95% CI: 1.406–1.872) were independent protective factors associated with preserved DLCO function.

### 3.8. Clinical Implications and Key Correlates of DLCO Impairment in COPD Patients

This schematic diagram summarizes the key clinical correlates, multi-system dysfunctions and clinical implications of progressive DLCO impairment in COPD patients based on the results of this cross-sectional study. DLCO impairment is a valuable complementary marker for COPD severity stratification, identifying a distinct high-risk phenotype with specific demographic, functional, comorbid and inflammatory characteristics, which could be used to guide targeted clinical interventions (Table 9).

## 4. Discussion

This cross-sectional study of 650 COPD patients systematically delineated the clinical, functional, and comorbid characteristics associated with varying degrees of DLCO impairment. The results demonstrate that progressive reduction in DLCO is linked to distinct demographic features, increased comorbidity burden, and multisystem functional decline. Given the cross-sectional design, these findings represent associations rather than causal relationships; they suggest that DLCO may be a useful phenotypic marker in COPD, but cannot establish prognostic or predictive value without longitudinal validation.

### 4.1. Demographic and Lifestyle Correlates of DLCO Impairment

Progressive DLCO impairment was strongly associated with male predominance (69.2% to 90.9%), advanced age (67.3 to 72.9 years), and lower BMI (23.9 to 20.0 kg/m^2^). This aligns with previous studies showing that male gender and aging are independent risk factors for DLCO decline in COPD, likely due to cumulative smoking exposure, occupational hazard exposure, and age-related physiological loss of alveolar surface area [14,15]. The inverse correlation between BMI and DLCO severity reflects the “COPD-cachexia” phenotype, where chronic inflammation, increased energy expenditure, and reduced nutritional intake synergistically impair both gas exchange function and nutritional status [16]. Notably, smoking prevalence increased with DLCO impairment (58.7% to 87.5%), while smoking pack-years showed no significant difference across groups—suggesting that smoking status (current/former) rather than cumulative exposure may be a stronger driver of DLCO decline, possibly due to individual susceptibility to tobacco-induced alveolar damage.

### 4.2. Clinical Symptoms, Signs, and Comorbidities

Shortness of breath (73.8% to 93.9%) and chest tightness (5.2% to 13.2%) were more prevalent in patients with severe DLCO impairment, consistent with the role of DLCO in gas exchange efficiency—reduced DLCO limits oxygen uptake during activity, exacerbating dyspnea [17]. Chest tightness, however, did not show a consistent linear trend, suggesting that symptom patterns are influenced by factors beyond diffusion impairment. The high incidence of barrel chest in the severe DLCO group (69.7%) reflects advanced emphysematous destruction, a key pathological substrate of DLCO decline [18].

Pulmonary tuberculosis prevalence increased with DLCO impairment, but the absolute numbers were small. This finding should be interpreted with caution and requires validation in larger cohorts [19]. Tuberculosis is known to cause parenchymal destruction and fibrotic changes that directly reduce DLCO; thus, reverse causation is at least as plausible as the hypothesis that DLCO impairment predisposes to tuberculosis. Similarly, although the prevalence of lung cancer and other tumors appeared numerically higher in the severe DLCO group, the comparisons were not statistically significant, and these observations should be regarded as exploratory signals [20]. Common comorbidities such as hypertension and diabetes showed no significant associations with DLCO, suggesting that they are driven by shared risk factors (e.g., aging) rather than direct DLCO-related pathways [21].

### 4.3. Functional and Systemic Inflammatory Correlates

All pulmonary function indices (FEV1%, FVC%, MEF series) declined progressively with DLCO impairment, with FEV1%pred dropping from 67.3% (normal DLCO) to 32.6% (severe DLCO). This confirms a synergistic relationship between airflow limitation and gas exchange dysfunction in COPD—chronic airway inflammation damages both small airways (reducing FEV1) and alveolar-capillary membranes (reducing DLCO) [22]. The decline in small airway indices (MEF75%, MEF50%) further underscores that DLCO impairment reflects diffuse respiratory tract involvement, not just alveolar damage [23].

Serological analyses revealed that DLCO decline was associated with systemic inflammation (elevated NLR and SII) and malnutrition (reduced albumin and hemoglobin). NLR and SII are validated markers of systemic inflammatory dysregulation in COPD, and their correlation with DLCO suggests that alveolar damage may amplify systemic inflammation via cytokine release [24]. Anemia and hypoalbuminemia, common in severe DLCO impairment, further exacerbate tissue hypoxia and functional decline, forming a vicious cycle [25].

Cardiac function analysis showed that severe DLCO impairment was associated with a small but statistically significant decline in EF%, though all values remained within the normal range, and the clinical relevance of this difference is uncertain. A non-significant trend toward higher PH prevalence (based on echocardiographic PASP > 30 mmHg) was observed in the severe DLCO group (*p* = 0.056). This finding should be considered hypothesis-generating, particularly given that echocardiography may overestimate true PH prevalence compared to right heart catheterization [26]. Chronic hypoxemia from DLCO decline induces pulmonary vasoconstriction and remodeling, increasing right ventricular afterload and predisposing to pulmonary hypertension [26]. This highlights the need for cardiac function monitoring in COPD patients with severe DLCO impairment.

### 4.4. Clinical Implications

Our findings support integrating DLCO into COPD assessment to improve risk stratification. Patients with severe DLCO impairment (DLCO%pred < 40%) are a high-risk subgroup characterized by male gender, advanced age, malnutrition, increased tuberculosis and cancer risk, and multi-system dysfunction. Targeted interventions—including nutritional support, anti-inflammatory therapy, tuberculosis screening, and cardiac function assessment—may improve outcomes in this population. Additionally, DLCO could complement FEV1 in clinical trials in identifying homogeneous patient subgroups for personalized therapy. Nevertheless, these implications are hypothesis-generating and require confirmation in prospective studies.

### 4.5. Limitations

This study has several limitations. First, its retrospective single-center design may introduce selection bias, and results may not be generalizable to other populations. Second, longitudinal data were unavailable to assess DLCO’s predictive value for long-term outcomes (e.g., acute exacerbations, mortality). Third, confounding factors such as occupational exposures, medication use (e.g., corticosteroids), and smoking cessation status were not fully adjusted for, which may influence DLCO and clinical outcomes. Fourth, DLCO was not corrected for hemoglobin levels, and VA/KCO were not measured, limiting physiological interpretation. Fifth, chest CT data were not quantitatively analyzed (e.g., emphysema extent), limiting insights into structural-functional correlations. Finally, the small number of patients in the severe DLCO group (n = 33) limits the precision of estimates for rare outcomes such as tuberculosis and lung cancer; findings based on single-digit counts should be interpreted with particular caution. Future multi-center prospective studies with longer follow-up and quantitative imaging are needed to validate these findings and explore DLCO-guided interventions.

## 5. Conclusions

In this cross-sectional study, DLCO impairment in COPD was associated with male sex, advanced age, lower BMI, higher smoking prevalence, increased tuberculosis history, worse lung function, systemic inflammation, and respiratory/cardiac dysfunction. These findings suggest that DLCO identifies a distinct clinical phenotype and may complement FEV_1_ in disease characterization. However, given the cross-sectional design and the single-center nature of the study, these findings should be considered hypothesis-generating. Prospective studies with longitudinal follow-up are required to determine whether DLCO provides independent prognostic value beyond conventional measures.

## Figures and Tables

**Table 1:** General Characteristics of COPD Patients with Different Degrees of Diffusion Impairment.

Variable	Normal DLCO (*n* = 172)	Mild DLCO (*n* = 256)	Moderate DLCO (*n* = 188)	Severe DLCO (*n* = 33)	*p* Value
Male, n (%)	119 (69.2)	190 (73.9)	150 (79.8)	30 (90.9)	0.018 *
Age, mean ± SD (years)	67.3 ± 8.97	70.3 ± 9.62	71.5 ± 8.72	72.9 ± 6.74	<0.001 **
BMI, median (IQR) (kg/m^2^)	23.9 (21.8, 26.6)	22.8 (21.0, 24.7)	21.5 (18.9, 23.6)	20.0 (16.8, 22.6)	<0.001 **
Smoking history, n (%)	101 (58.7)	173 (67.6)	138 (74.2)	28 (87.5)	0.001 *
Smoking pack-years, median (IQR)	37.5 (25, 40)	40 (20, 50)	40 (30, 50)	30 (20, 50)	0.169
Symptoms, n (%)					
Cough	142 (82.6)	219 (85.5)	163 (86.7)	32 (97.0)	0.175
Expectoration	132 (76.7)	206 (80.5)	156 (83.0)	31 (93.9)	0.106
Shortness of breath	127 (73.8)	195 (76.2)	152 (80.9)	31 (93.9)	0.047 *
Chest tightness	9 (5.2)	34 (13.2)	15 (8.0)	1 (3.0)	0.017 *
Fatigue	86(50.0)	115(44.9)	92(48.9)	19(57.6)	0.471
Signs, n (%)					
Barrel chest	62 (36.0)	97 (37.7)	80 (42.6)	23 (69.7)	0.003 *
Wheezes	50 (29.1)	46 (17.9)	43 (22.9)	12 (36.4)	0.014 *
Moist rales	51 (29.7)	92 (35.8)	66 (35.1)	10 (30.3)	0.551

* *p* < 0.05, ** *p* < 0.01; IQR = interquartile range; BMI = body mass index.

**Table 2:** Analysis of Comorbidities in COPD Patients with Different Degrees of Diffusion Impairment.

Comorbidity	Normal DLCO (*n* = 172)	Mild DLCO (*n* = 256)	Moderate DLCO (*n* = 188)	Severe DLCO (*n* = 33)	*p* Value
Underlying diseases, *n* (%)					
Hypertension	63 (36.6)	92 (35.9)	72 (38.3)	14 (42.4)	0.878
Diabetes mellitus	27 (15.7)	38 (14.8)	26 (13.8)	4 (12.1)	0.934
Coronary heart disease	18 (10.5)	37 (14.5)	27 (14.4)	3 (9.1)	0.534
Pulmonary tuberculosis	1 (0.58)	7 (2.73)	6 (3.19)	3 (9.09)	0.037 *
Thyroid diseases	10 (5.81)	12 (4.69)	5 (2.66)	1 (3.03)	0.494
Malignant tumors, *n* (%)					
Confirmed lung cancer	6 (3.49)	17 (6.61)	12 (6.38)	4 (12.12)	0.227
Other tumors	8 (4.65)	9 (3.52)	11 (5.85)	3 (9.09)	0.431

* *p* < 0.05.

**Table 3:** Pulmonary Function Indices of COPD Patients with Different Degrees of Diffusion Impairment.

Parameter	Normal DLCO (*n* = 172)	Mild DLCO (*n* = 256)	Moderate DLCO (*n* = 188)	Severe DLCO (*n* = 33)	*p* Value
VC%pred	87.5 (77.3, 99.5)	84.9 (71.9, 98.3)	77.6 (64.3, 88.3)	64.3 (56.9, 76.7)	<0.001 **
FVC%pred	87.7 (78.5, 102.9)	86.9 (72.9, 100.7)	79.2 (66.2, 90.3)	64.1 (58.3, 76.8)	<0.001 **
FEV1%pred	67.3 (52.7, 84.4)	61.4 (45.7, 78.1)	51.0 (35.8, 65.1)	32.6 (23.5, 51.7)	<0.001 **
DLCO%pred	90.6 (85.4, 98.6)	69.6 (64.8, 74.4)	50.1 (45.0, 55.7)	33.0 (29.9, 35.9)	<0.001 **
MEF75%pred	40.2 (24.4, 61.5)	30.4 (17.5, 50.3)	22.4 (11.2, 41.6)	9.4 (6.6, 22.7)	<0.001 **
MEF50%pred	26.4 (17.1, 37.3)	19.5 (12.2, 32.5)	14.9 (8.9, 26.6)	8.0 (5.7, 16.5)	<0.001 **
MEF25%pred	22.5 (16.9, 29.3)	18.8 (13.8, 29.5)	18.6 (12.6, 28.7)	15.8 (11.4, 25.3)	0.003 *
MMEF%pred	23.7 (16.1, 33.7)	18.7 (12.5, 30.9)	15.5 (9.7, 25.5)	9.7 (7.1, 16.7)	<0.001 **

* *p* < 0.05, ** *p* < 0.01; VC = vital capacity, FVC = forced vital capacity, FEV1 = forced expiratory volume in 1 s, DLCO = diffusing capacity of carbon monoxide, MEF = maximum expiratory flow, MMEF = maximal mid-expiratory flow.

**Table 4:** Serological Indices of COPD Patients with Different Degrees of Diffusion Impairment.

Variable	Normal DLCO (*n* = 172)	Mild DLCO (*n* = 256)	Moderate DLCO (*n* = 188)	Severe DLCO (*n* = 33)	*p* Value
Blood routine					
White blood cell count (10^9^/L), median (Q1, Q3)	6.86 (5.63, 9.14)	6.92 (5.50, 8.83)	7.68 (6.09, 9.41)	6.76 (5.35, 9.55)	0.084
Hemoglobin (g/L), median (Q1, Q3)	140.0 (131.0, 149.0)	130.0 (117.0, 140.0)	130.0 (117.0, 142.0)	126.5 (114.5, 139.8)	<0.001 **
Anemia, *n* (%)	9 (5.2)	40 (15.6)	43 (22.9)	10 (30.3)	<0.001 **
Platelet count (10^9^/L), median (Q1, Q3)	214.00 (178, 247)	216.00 (168, 272)	211.50 (160.75, 270.50)	209.50 (156, 251.25)	0.819
Neutrophil count (10^9^/L), median (Q1, Q3)	4.94 (3.94, 6.21)	4.82 (3.62, 6.48)	5.43 (4.26, 7.43)	5.40 (3.28, 7.25)	0.056
Lymphocyte count (10^9^/L), median (Q1, Q3)	1.34 (1.06, 1.88)	1.27 (0.97, 1.57)	1.31 (0.97, 1.87)	1.02 (0.80, 1.38)	0.003 *
NLR, median (Q1, Q3)	3.9 (2.6, 4.7)	4.0 (2.7, 5.7)	4.2 (3.1, 6.3)	5.4 (2.9, 7.5)	0.002 *
SII, median (Q1, Q3)	778.3 (489.6, 1141.5)	861.4 (490.8, 1229.9)	882.6 (551.3, 1309.0)	1161.1 (426.0, 1806.0)	0.010 *
Albumin (g/L), median (Q1, Q3)	41.6 (39.8, 43.9)	40.1 (38.1, 42.4)	39.2 (36.8, 41.6)	39.1 (37.3, 41.8)	<0.001 **
Hypoalbuminemia, *n* (%)	7/140 (5.00)	6/184 (3.26)	17/155 (10.97)	2/31 (6.45)	0.001 *
Arterial blood gas analysis					
pH, median (Q1, Q3)	7.42 (7.40, 7.44)	7.42 (7.40, 7.45)	7.43 (7.40, 7.45)	7.40 (7.38, 7.45)	0.198
PaO_2_ (mmHg), median (Q1, Q3)	71.00 (64.40, 81.00)	70.30 (63.30, 79.00)	66.55 (60.70, 73.93)	68.70 (57.88, 82.68)	0.219
PaCO_2_ (mmHg), median (Q1, Q3)	39.60 (37.70, 43.30)	39.90 (37.00, 43.40)	39.90 (35.88, 43.85)	40.90 (37.82, 46.35)	0.400
Oxygen saturation (%), median (Q1, Q3)	95.8 (93.6, 97.0)	95.0 (92.7, 96.2)	94.3 (92.0, 95.3)	93.8 (84.9, 96.6)	0.018 *
Hypoxemia, *n* (%)	92/127 (72.44)	130/173 (75.14)	117/151 (77.48)	21/30 (70.00)	0.013 *
Respiratory failure, *n* (%)	8 (6.3)	24 (13.9)	25 (16.6)	9 (30.0)	<0.001 **
Type I respiratory failure, *n* (%)	7 (87.5)	18 (75.00)	19 (76.00)	7 (77.78)	<0.001 **
Type II respiratory failure, *n* (%)	1 (12.5)	6 (25.00)	6 (24.00)	2 (22.22)	0.004 *

* *p* < 0.05, ** *p* < 0.01; NLR = neutrophil-to-lymphocyte ratio; SII = systemic immune-inflammation index; PaO_2_: Arterial oxygen partial pressure; PaCO_2_: Arterial carbon dioxide partial pressure. Hypoalbuminemia: Albumin < 35 g/L; Anemia: < 120 g/L (Male), < 110 g/L (Female); Hypoxemia: PaO_2_ < 80 mmHg; Type I respiratory failure: PaO_2_ < 60 mmHg with normal or decreased PaCO_2_; Type II respiratory failure: PaO_2_ < 60 mmHg and PaCO_2_ > 50 mmHg.

**Table 5:** Echocardiographic Parameters of COPD Patients with Different Degrees of Diffusion Impairment.

Parameter	Normal DLCO (*n* = 87)	Mild DLCO (*n* = 116)	Moderate DLCO (*n* = 104)	Severe DLCO (*n* = 18)	*p* Value
EF%, mean ± SD	61.2 ± 5.0	61.9 ± 4.3	60.1 ± 5.2	59.1 ± 4.0	0.012 *
Right ventricular outflow tract diameter, mean ± SD (mm)	26.8 ± 2.9	26.3 ± 2.7	27.4 ± 3.3	26.3 ± 3.2	0.041 *
PASP (mmHg), median (Q1, Q3)	38.0 (37.0, 48.0)	39.0 (30.8, 50.8)	37.0 (32.0, 46.0)	40.6 (30.0, 49.0)	0.411
PH, n (%)	13 (14.9)	14 (12.1)	25 (24.0)	5 (27.8)	0.056

* *p* < 0.05; EF = ejection fraction; PASP: Pulmonary arterial systolic pressure; PH = Pulmonary hypertension: PASP > 30 mmHg.

**Table 6:** Analysis of Chest Imaging Characteristics in COPD Patients with Varying Degrees of DLCO Impairment.

Parameter	Normal DLCO (*n* = 172)	Mild DLCO (*n* = 256)	Moderate DLCO (*n* = 188)	Severe DLCO (*n* = 33)	*p* Value
**Emphysema type**					
Centrilobular	82 (47.7)	131 (51.0)	122 (64.9)	25 (75.8)	<0.001 **
Panlobular	0 (0.0)	3 (1.2)	5 (2.7)	4 (12.1)	0.001 *
Paraseptal	6 (3.5)	13 (5.1)	14 (7.4)	0 (0.0)	0.183
**Fibrotic features**					
Honeycombing	0 (0.0)	1 (0.4)	6 (3.2)	1 (3.0)	0.010 *
Reticulation	0 (0.0)	7 (2.7)	11 (5.9)	1 (3.0)	0.012 *
Cysts	4 (2.3)	7 (2.7)	7 (3.7)	1 (3.0)	0.878
Traction bronchiectasis	2 (1.2)	2 (0.8)	12 (6.4)	4 (12.1)	<0.001 **
Subpleural line	2 (1.2)	1 (0.4)	2 (1.1)	0 (0.0)	0.705
**Other findings**					
Bronchiectasis	46 (26.7)	63 (24.5)	51 (27.1)	5 (15.2)	0.494
Bullae	23 (13.4)	52 (20.2)	62 (33.0)	15 (45.5)	<0.001 **
Bronchiolitis	17 (9.9)	11 (4.3)	7 (3.7)	0 (0.0)	0.016 *
Air trapping	8 (4.7)	2 (0.8)	0 (0.0)	1 (3.0)	0.002 *

Data are presented as *n* (%). * *p* < 0.05, ** *p* < 0.01.

**Table 7:** Correlation Between DLCO%pred and Clinical Indices.

Index	Correlation Coefficient (r)	*p* Value
FEV1%pred	0.394	<0.001 **
FVC%pred	0.374	<0.001 **
VC%pred	0.375	<0.001 **
Oxygen saturation (%)	0.151	<0.001 **
Hemoglobin (g/L)	0.182	<0.001 **
Lymphocyte count (10^9^/L)	0.130	<0.001 **
Neutrophil percentage (%)	−0.146	<0.001 **
NLR	−0.165	<0.001 **
SII	−0.149	<0.001 **

** *p* < 0.01; NLR = neutrophil-to-lymphocyte ratio, SII = systemic immune-inflammation index.

**Table 8:** Logistic regression analysis of factors affecting DLCO function.

Variable	β Coefficient	*p* Value	OR (Exp(β))	95% CI for OR
Age (years)	–0.033	0.013 **	0.968	0.943–0.993
BMI (kg/m^2^)	0.168	<0.001 **	1.183	1.108–1.263
Oxygen saturation (%)	0.120	0.002 **	1.128	1.043–1.219
Hemoglobin (g/dL)	0.484	<0.001 **	1.622	1.406–1.872

Hemoglobin was expressed in g/dL to align with the Cotes correction formula. ** *p* < 0.05.

**Table 9:** Clinical Implications and Key Correlates of DLCO Impairment in COPD Patients. ↑ indicates an increasing trend; ↓ indicates a decreasing trend.

Progressive DLCO Impairment (Normal→Mild→Moderate→Severe)	Key Clinical Correlates & Multi-System Dysfunctions	Clinical Implications & Targeted Interventions
DLCO%pred ≥80% 60–79% 40–59% <40%	**Demographic/Lifestyle** ↑ Male proportion (69.2→90.9%) ↑ Age (67.3→72.9 yrs) ↓ BMI (23.9→20.0 kg/m^2^) ↑ Smoking prevalence (58.7→87.5%) ↑ Smoking pack-years (37.5→45.0)	**Risk Stratification** - DLCO as a complementary marker to FEV1/GOLD stage - Severe DLCO impairment (<40%) = high-risk phenotype
	**Pulmonary Function** ↓ All spirometric indices (FEV1%pred:67.3→32.6%) ↓ Small airway function (MEF75/50/25) ↑ Respiratory failure (6.3→30.0%, predominantly Type I)	**Pulmonary Management** - Intensified lung function monitoring - Oxygen therapy for hypoxemia/respiratory failure
	**Systemic Status** ↑ Systemic inflammation (NLR/SII) ↓ Hemoglobin/Albumin ↑ Anemia prevalence (5.2→30.3%)	**Systemic Support** - Nutritional supplementation for malnutrition/anemia - Anti-inflammatory therapy for elevated NLR/SII
	**Cardiac Complications** ↓ Left ventricular ejection fraction (61.2→59.1%) ↑ Pulmonary hypertension risk	**Cardiac Monitoring** - Routine echocardiography for severe DLCO impairment - Early intervention for pulmonary vascular dysfunction
	**Comorbidities** ↑ Pulmonary tuberculosis (0.58→9.09%) ↑ Trend of lung cancer/other tumors	**Comorbidity Screening** - Regular tuberculosis screening - Cancer surveillance for high-risk patients

## Data Availability

The data presented in this study are available on request from the corresponding author due to ethical and privacy restrictions related to the retrospective use of clinical patient data from the electronic medical record system.

## References

[1] Global Initiative for Chronic Obstructive Lung Disease Global Strategy for the Diagnosis, Management, and Prevention of Chronic Obstructive Pulmonary Disease (2025 Report). https://goldcopd.org/.

[2] Han M.K., Agusti A., Calverley P.M., Celli B.R., Criner G., Curtis J.L., Fabbri L.M., Goldin J.G., Jones P.W., MacNee W. (2020). Chronic obstructive pulmonary disease phenotypes: The future of COPD. Am. J. Respir. Crit. Care Med..

[3] Jones P.W. (2009). Health status and the spiral of decline. COPD J. Chronic Obstr. Pulm. Dis..

[4] Han M.L.K., Muellerova H., Curran-Everett D., Dransfield M.T., Washko G.R., Regan E.A., Bowler R.P., Beaty T.H., Hokanson J.E., Lynch D.A. (2013). GOLD 2011 disease severity classification in COPDGene: A prospective cohort study. Lancet Respir. Med..

[5] Balasubramanian A., Putcha N., MacIntyre N.R., Jensen R.L., Kinney G., Stringer W.W., Hersh C.P., Bowler R.P., Casaburi R., Han M.K. (2023). Diffusing capacity and mortality in chronic obstructive pulmonary disease. Ann. Am. Thorac. Soc..

[6] Choi J., Sim J.K., Oh J.Y., Lee Y.S., Hur G.Y., Lee S.Y., Shim J.J., Rhee C.K., Min K.H. (2021). Prognostic marker for severe acute exacerbation of chronic obstructive pulmonary disease: Analysis of diffusing capacity of the lung for carbon monoxide (DLCO) and forced expiratory volume in one second (FEV1). BMC Pulm. Med..

[7] Devalla L., Ghewade B., Jadhav U., Annareddy S. (2024). Resolving the Complexity: A Comprehensive Review on Carbon Monoxide Diffusion Capacity in Chronic Obstructive Pulmonary Disease Patients. Cureus.

[8] Wan P.F., Xu J., Chen H.P., Hu M.D. (2021). Clinical characteristics and risk factors of chronic obstructive pulmonary disease with tuberculosis. Chin. J. Lung Dis. Electron. Ed..

[9] Rose L., Prins K.W., Archer S.L., Pritzker M., Weir E.K., Misialek J.R., Thenappan T. (2019). Survival in pulmonary hypertension due to chronic lung disease: Influence of low diffusion capacity of the lungs for carbon monoxide. J. Heart Lung Transplant..

[10] Casanova C., Gonzalez-Dávila E., Martínez-Gonzalez C., Cosio B.G., Fuster A., Feu N., Solanes I., Cabrera C., Marin J.M., Balcells E. (2021). Natural course of the diffusing capacity of the lungs for carbon monoxide in COPD: Importance of sex. Chest.

[11] Balasubramanian A., MacIntyre N.R., Henderson R.J., Jensen R.L., Kinney G., Stringer W.W., Hersh C.P., Bowler R.P., Casaburi R., Han M.K. (2019). Diffusing capacity of carbon monoxide in assessment of COPD. Chest.

[12] Wanger J., Clausen J.L., Coates A., Pedersen O.F., Brusasco V., Burgos F., Casaburi R., Crapo R., Enright P., van der Grinten C. (2005). ATS/ERS Task Force: Standardisation of lung function testing. Standardisation of the measurement of lung volumes. Eur. Respir. J..

[13] Miller M.R., Hankinson J., Brusasco V., Burgos F., Casaburi R., Coates A., Crapo R., Enright P., Van Der Grinten C.P.M., Gustafsson P. (2005). Standardisation of spirometry. Eur. Respir. J..

[14] Blomberg A., Torén K., Liv P., Granåsen G., Andersson A., Behndig A., Bergström G., Brandberg J., Caidahl K., Cederlund K. (2024). Chronic Airflow Limitation, Emphysema, and Impaired Diffusing Capacity in Relation to Smoking Habits in a Swedish Middle-aged Population. Ann. Am. Thorac. Soc..

[15] Hu Y.X., Zhang L., Fan J.X. (2020). Analysis of the levels of related functional indexes and risk factors of chronic obstructive pulmonary disease in the elderly patients. Pract. Geriatr..

[16] Priya K., Vadivelu S., Abraham E.A., Verma G., PG A., Mounika P., Elango R. (2025). Correlation Between Chronic Obstructive Pulmonary Disease Severity and Nutritional Status: A Cross-Sectional Study from a Tertiary Care Center in South India. Cureus.

[17] Elbehairy A.F., O’Donnell C.D., Elhameed A.A., Vincent S.G., Milne K.M., James M.D., Webb K.A., Neder J.A., O’donnell D.E., Canadian Respiratory Research Network (2019). Low resting diffusion capacity, dyspnea, and exercise intolerance in chronic obstructive pulmonary disease. J. Appl. Physiol..

[18] Criner G.N., Hatt C.R., Galbán C.J., Kazerooni E.A., Lynch D.A., McCormack M.C., Casaburi R., MacIntyre N.R., Make B.J., Martinez F.J. (2019). Relationship between diffusion capacity and small airway abnormality in COPDGene. Respir. Res..

[19] Arora V.K., Rajpal S. (2025). Tuberculosis and COPD: A Multimorbidity Syndrome. Indian J. Tuberc..

[20] Tang J., Ramiscabrer D., Curull V., Wang X., Qin L., Mateu-Jiménez M., Duran X., Pijuan L., Rodríguez-Fuster A., Espases R.A. (2020). Immune Cell Subtypes and Cytokines in Lung Tumor Microenvironment: Influence of COPD. Cancers.

[21] Gale N.S., Albarrati A.M., Munnery M.M., Mcdonnell B.J., Benson V.S., Tal-Singer R.M., Cockcroft J.R., Shale D.J. (2019). Aortic pulse wave velocity as a measure of cardiovascular risk in chronic obstructive pulmonary disease: Two-year follow-up data from the ARCADE study. Medicina.

[22] Agustí A., Celli B.R., Criner G.J., Halpin D., Anzueto A., Barnes P., Bourbeau J., Han M.K., Martinez F.J., de Oca M.M. (2023). Global initiative for chronic obstructive lung disease 2023 report: GOLD executive summary. Eur. Respir. J..

[23] Tanabe N., Rhee C.K., Sato S., Muro S., Shima H., Tanimura K., Jung K.S., Yoo K.H., Hirai T. (2020). Disproportionally Impaired Diffusion Capacity Relative to Airflow Limitation in COPD. COPD J. Chronic Obstr. Pulm. Dis..

[24] Song Y., Bai X., Ma J. (2024). The association of systemic immune-inflammation index with lung function, risk of COPD, and COPD severity: A population-based study. PLoS ONE.

[25] Collins P.F., Elia M., Stratton R.J. (2013). Nutritional support and functional capacity in chronic obstructive pulmonary disease: A systematic review and meta-analysis. Respirology.

[26] Diamanti E., Karava V., Yerly P., Aubert J.D. (2021). Carbon Monoxide Diffusion Capacity as a Severity Marker in Pulmonary Hypertension. J. Clin. Med..

